# Experimental Demonstration of a Visible Light Communications System Based on Binary Frequency-Shift Keying Modulation: A New Step toward Improved Noise Resilience

**DOI:** 10.3390/s23115001

**Published:** 2023-05-23

**Authors:** Cătălin Beguni, Adrian Done, Alin-Mihai Căilean, Sebastian-Andrei Avătămăniței, Eduard Zadobrischi

**Affiliations:** 1 Integrated Center for Research, Development and Innovation in Advanced Materials, Nanotechnologies and Distributed Systems for Fabrication and Control, Stefan cel Mare University of Suceava, 720229 Suceava, Romania; sebastian.avatamanitei@usm.ro (S.-A.A.); eduard.zadobrischi@usm.ro (E.Z.); 2 Department of Computers, Electronics and Automation, Stefan cel Mare University of Suceava, 720229 Suceava, Romania; adone@eed.usv.ro; 3 Systems Engineering Laboratory of Versailles, Paris-Saclay University, UVSQ, 78140 Vélizy, France

**Keywords:** binary frequency-shift keying modulation, BFSK, frequency modulation, noise resilience, optical communications, optical wireless communications, visible light communication, VLC

## Abstract

Visible light communications (VLC) are an emerging technology that is increasingly demonstrating its ability to provide wireless communications in areas where radio frequency (RF) technology might have some limitations. Therefore, VLC systems offer possible answers to various applications in outdoor conditions, such as in the road traffic safety domain, or even inside large buildings, such as in indoor positioning applications for blind people. Nevertheless, several challenges must still be addressed in order to obtain a fully reliable solution. One of the most important challenges is focused on further improving the immunity to optical noise. Different from most works, where on–off keying (OOK) modulation and Manchester coding have been the preferred choices, this article proposes a prototype based on a binary frequency-shift keying (BFSK) modulation and non-return-to-zero (NRZ) coding, for which the resilience to noise is compared to that of a standard OOK VLC system. The experimental results showed an optical noise resilience improvement of 25% in direct exposure to incandescent light sources. The VLC system using BFSK modulation was able to maintain a maximum noise irradiance of 3500 µW/cm^2^ as compared with 2800 µW/cm^2^ for the OOK modulation, and an improvement of almost 20% in indirect exposure to the incandescent light sources. The VLC system with BFSK modulation was able to maintain the active link in an equivalent maximum noise irradiance of 65,000 µW/cm^2^, as opposed to the equivalent 54,000 µW/cm^2^ for the OOK modulation. Based on these results, one can see that based on a proper system design, VLC systems are able to provide impressive resilience to optical noise.

## 1. Introduction

Visible light communications (VLC) are a branch of optical wireless communications (OWC) which use the visible light spectrum (380–780 nm) for simultaneous illumination and data communication [1,2]. To fulfil this aim, the VLC technology uses fast switching solid-state lighting (SSL) sources such as light emitting diodes (LEDs), organic LEDs (OLEDs), or future-generation laser lighting devices. As the VLC technology has further developed, additional functions have been integrated, enabling such systems to also provide distance measurements and relative positioning [3,4,5]. In terms of advantages, VLC benefits from the fact that the technology is developing on top of a preexisting lighting infrastructure, generating a low implementation cost, wide area coverage, and high potential for rapid deployment. In addition, the fact that VLC is an energy-efficient multi-purpose technology makes it extremely promising in the current context in which human society is making the transition toward an energy-efficient society [6]. On the other hand, the development of the VLC technology is also motivated by the fact that human society is manifesting an unpreceded demand for wireless communication technologies [7,8]. Different from previous times in which wireless technologies were mostly used in traditional communication purposes, the new paradigm envisions the integration of these technologies and of the VLC technology, in particular, in intelligent and autonomous vehicles [9,10], high data rate indoor applications for internet and multimedia applications [11], or multi-purpose Internet of Things (IoT) applications [12,13]. Therefore, although VLC is a relatively new technology, its performances have reached a relatively high maturity level. Thus, indoor VLC prototypes have reached data rates of few tens of gigabits per second [11], are able to provide centimeter-level positioning accuracy [14], and can manage multiple users simultaneously [15]. On the other hand, vehicular VLC prototypes have reached almost 200 m communication ranges [16,17], have significantly improved optical noise resilience [18,19,20], and have provided latencies lower than 100 ms [19,21], while demonstrating their ability to be used in real traffic safety applications [22,23,24]. Although these results are rather impressive from many perspectives, continuous technology improvement is mandatory.

A specific challenge for VLC systems is related to their ability to provide high reliability even in the presence of optical noise sources [9]. Although significant progress has been made in this direction, this challenge is still open due to the intrinsic characteristics of VLC. A VLC receiver is usually based on a PIN photodiode connected in a transimpedance circuit. The PIN photodiode generates an electrical current which is directly proportional to the incident light. Nevertheless, in many cases, the VLC receiver is exposed to other sources of light, not just to the VLC transmitter. In vehicular applications, the VLC receiver can be exposed to multi-user interferences (MUI) [25,26], but it can also be exposed to a multitude of other light sources (artificial and natural) [9]. Among all optical noise sources, sunlight is considered to be the most disruptive, as it can reach intensities that can go above 65,000 µW/cm^2^. On the other hand, the intensities of the data signal can vary from few tens of µW/cm^2^ in standard indoor applications and go as low as a few nW/cm^2^ in vehicular VLC applications.

In this context, this article proposes a new concept of VLC data transmission based on binary frequency-shift keying (BFSK) modulation. The proposed BFSK concept is briefly described, implemented in a VLC system, and evaluated in laboratory conditions. To emphasize the improved noise resilience, the prototype performances are compared to the ones of a standard VLC prototype based on on–off keying (OOK) modulation and Manchester coding. The experimental results have demonstrated the high potential of the BFSK modulation, proving that such a technique enables the VLC prototype to work under the exposure of a light source, having an irradiance that can reach as high as 65,000 µW/cm^2^. Different from most of the existing works which propose the evaluation of different modulations in VLC applications, this work provides a very strong hardware component, with a specific prototype being designed, implemented, and experimentally evaluated. Consequently, although the BFSK modulation is not a novel concept, the novelty of this work is provided by the following:

✓ i. Analyzing the opportunity of using BFSK modulation in VLC applications;
✓ ii. Developing and implementing a BFSK VLC prototype;
✓ iii. Experimentally evaluating the BFSK VLC prototype while comparing its performances with the ones of a classical VLC solution based on OOK modulation;
✓ iv. Demonstrating experimentally the fact that BFSK provides enhanced noise resilience in VLC applications.

On the other hand, as this prototype is still in testing, certain data rate issues must be acknowledged.

The rest of this article is organized as follows. Section 2 aims to present some of the existing techniques that are used to improve the VLC receivers’ optical noise resilience. Next, Section 3 provides a brief description of the BFSK VLC concept, whereas Section 4 debates the aspects related to the hardware implementation of the VLC prototype. Following this, Section 5 presents the setup for the VLC experiments and delivers the experimental results. In the end, Section 6 delivers the conclusions and provides a brief discussion on the perspectives of this work.

## 2. State of the Art in Noise-Resilient Visible Light Communications Systems

Due to their intrinsic characteristics, VLCs have been considered as a rather reduced resilience wireless communication technology. Nevertheless, due to many unique advantages, the research community has continued to work on this issue, and, thus, current VLC systems have managed to make significant progress in this direction. It should be emphasized that although noise resilience is crucial for vehicular VLC systems [9,10], this issue is vital for indoor VLC systems as well [27]. On the other hand, the progress toward optical-noise-resilient VLC systems has been mostly driven by vehicular VLC systems developers.

In the first years, noise resilience in VLC applications was achieved mostly by narrowing the VLC receiver field-of-view (FoV). Thus, some of the first VLC systems achieved noise resilience by using a narrow FoV as low as 0.5–2° [28,29]. The results confirm the effectiveness of this method in terms of enhanced noise resilience. On the downside, although the effect of noise sources is mitigated, the narrow FoV significantly affects the mobility of the system. An extended study which shows the dependency between the VLC receiver FoV and noise resilience is available in [30]. A different approach to enhance noise resilience is found in [31]. In this case, authors use an improved modulation technique based on direct-sequence spread spectrum (DSSS) and sequence inverse keying (SIK) to ensure improved noise resilience, while sacrificing the data rate. The authors of [32] increase noise resilience by trading the communication range in turn. In this case, the amplification factor of the transimpedance circuit is established at a limit that prevents photodiode saturation, while insufficient signal amplification is affecting the communication range.

The high level of optical interference associated with VLC applications is also acknowledged in the IEEE 802.15.7 standard for optical communications using visible light. To deal with this problem, the standard specifies Reed–Solomon (RS) and convolutional codes (CC) as a forward error correction (FEC) technique [33]. In accordance with the application and the associated noise level, different combinations of these codes are applied, ensuring a signal-to-noise (SNR) improvement that can go up to 10 dB. On the downside, FEC significantly affects the data rate [33]. For example, depending on the applied FEC, the data rate of PHY I applications using OOK and Manchester coding can range between 11 and 100 kb/s.

Another approach to mitigate the effect of optical noise source in VLC applications is based on optical filter usage. Optical filters eliminate part of the unwanted optical spectral components leaving only the desired optical components, ensuring a significant SNR enhancement [16,17,23,24]. A liquid crystal display (LCD)-based optical filtering solution is proposed in [34,35].

Based on the improved filtering performance associated with digital filters, the authors of [36] proposed a digital signal processing (DSP) VLC architecture to mitigate the effect of optical noise and to ensure improved flexibility in signal processing. Another example of DSP usage in a software-defined radio (SDR) receiver is found in [20]. The experimental results showed that DSP can provide a 40 dB noise margin improvement, enabling the system to maintain the link activity even during sunlight exposure, or under direct 100 Hz noise generated by indoor lighting. In addition to the traditional DSP technique utilization in VLC, we can witness a current paradigm shift toward artificial intelligence (AI), deep learning (DL) and neural networks (NN) [37,38]. The integration of these techniques on top of the traditional DSP techniques can enable VLC systems to better adapt to the communication channel [37,38].

The authors of [27] addressed the problems associated with strong sunlight exposure based on the use of a narrow band-pass optical filter and orthogonal frequency-division multiplexing (OFDM) modulation. Thus, these techniques enabled them to provide a 1 Gb/s data rate under a 50,350 lux noise exposure, for a 14 cm range.

In [18,19], a new concept of a logarithmic transimpedance circuit is proposed and experimentally evaluated. The intensive experimental evaluation has shown that the logarithmic transimpedance solution prevents photoelement saturation, while providing improved noise resilience, and an extended dynamic range.

A new method for optical noise suppression in VLC is demonstrated in [39]. This concept uses polarized light to transmit differential signals over adjacent channels. At the receiver side, differential amplification and polarization are used to reject any common-mode optical noise, providing optical-noise-tolerant VLC.

Relay-assisted VLC represents another solution for noise resilience improvement. This technique envisions that network nodes that are able to receive certain messages retransmit them on different paths for other network nodes that are exposed to optical noise or that are misaligned [40,41].

In addition to the concepts presented above, numerous other solutions focused on optical noise suppression in VLC applications are found in the literature [42,43]. Nevertheless, most of these concepts are demonstrated only by simulation means. Thus, solutions and approaches based on better modulations and coding techniques are also developed, analyzed, and optimized. Based on its ability to improve data rate, OFDM is considered as highly promising in future VLC applications [44]. Here [44], the authors propose a DC optical OFDM pulse-position modulation which is evaluated against a DC optical OFDM pulse-width modulation (PWM), showing lower SNR requirements. Acknowledging the benefits of OFDM, and also the fact that its use can generate high peak-to-average power ratio (PAPR), the authors of [45] propose and evaluate a PAPR reducing algorithm based on a Vandermonde-like matrix combined with a Gaussian matrix, providing a 11–34% PAPR reduction with respect to other methods. The authors of [46] focus on the use of the VLC technology in an industrial IoT scenario. After using ray tracing to demonstrate that this environment is more challenging than the standard indoor utilization scenario, they propose a link adaptation mechanism aimed at reducing the effects of multipath interference. Again, DC OFDM is considered as a suitable modulation technique. It should be emphasized that [44,45,46], as well as most of the works that are focused on different types of modulation evaluation are based on simulations, whereas the VLC channel model provided in [47] remains the basis for most of today’s channel models. Different from FSK-based modulation such as OFDM, the work focused on phase-shift keying (PSK) modulation is rather limited, with only few such papers being available [48,49]. Despite some benefits [48,49], identifying the exact phase in optical noise conditions might be problematic. In such conditions, high frequency noise that is generated (i.e., shot noise and thermal noise) can introduce signal distortions which might pose decoding problems and lower bit error ratio (BER), especially when higher order PSK schemes are envisioned. Otherwise, 2-PSK acts like the classical Manchester coding (i.e., also known as bi-phase modulation). On the other hand, PSK modulation requires adequate synchronization in phase and frequency between the VLC emitter and the VLC receiver. Nevertheless, in decentralized architectures, asynchronous links are generally better suited.

In light of the above-mentioned works, one can see that important progress in the area of VLC noise resilience has been made. Nevertheless, due to the high impact of optical noise on the performance of VLC systems, additional investigations are more than welcome. In the end, one can expect that full noise resilience will be achieved based on the integration of a mix of solutions, some focused on improved modulation and coding techniques, and others on optical solutions based on lens and optical filters, enhanced hardware design of the transimpedance circuit, better signal processing, improved decoding techniques, and FEC use.

In the end, it should be mentioned that in outdoor scenarios, weather phenomena such as snowfall, rain, or fog also significantly affect the VLC channel [9,50]. In their cases, the light passage is affected by a combination of reflection, refraction, and scattering which limit the useful signal reaching the VLC receiver, affecting in turn the SNR and the link performance [19,50]. Nevertheless, as mentioned in the motivation of this work, this article is focused on the issues generated by optical interference, which is generated by light sources, and, thus, dealing with such weather phenomena is not within the purpose of this work.

## 3. Binary Frequency-Shift Keying Visible Light Communications Concept Presentation

Binary frequency-shift keying modulation is a subtype of frequency shift keying modulation where digital data are coded in such a way that for every bit, i.e., a “0” or a “1”, a discrete frequency is sent. This means that a sine frequency noted *f*_1_ is allocated to the “0”-bit, and another different sine frequency noted *f*_2_ is allocated to the “1”-bit, which are referred to in the literature as “space frequency” and “mark frequency”, respectively. In this regard, the frequency carrier for BFSK can be defined as an average for the mark and space frequencies:

(1) $$f_{c}=\frac{f_{1}+f_{2}}{2},$$

and the deviation of the frequency can be defined as

(2) $${∆}_{f}=\frac{f_{2}-f_{1}}{2}.$$

According to Carson’s rule, the bandwidth of BFSK modulation can be calculated based on the baud rate *R_b_* of the data stream [51]:

(3) $$B=2({∆}_{f}+R_{b}).$$

The minimum bandwidth necessary for a reliable orthogonal demodulation is obtained when the frequency separation is 1/2*T_s_*, where *T_s_* is the symbol period, but this can be achieved when the received mark and space carriers are phase aligned (i.e., coherent demodulation). The condition of coherence is hard to implement in VLC systems, so the non-coherent demodulation is preferred, which means that the frequency separation must be *R_b_* = *1/T_s_* for orthogonal demodulation, meaning that a bandwidth of at least 4*R_b_* would be needed in this case. Anyway, regardless of the frequency separation, which only gives us the total bandwidth for BFSK modulation, in order to properly decode the signal, the necessary band-pass filters for each carrier would only be *2R_b_*. For a non-return-to-zero (NRZ) coding, the symbol period at 10 kbps is 100 ns; so, the necessary bandwidth for the BFSK modulation would be 20 kHz.

In order to have a sense of the behavior of this prototype with BFSK modulation, the basic idea is to compare it with the more common OOK modulation with Manchester coding. The bandwidth for a Manchester signal can be deducted from the power spectral density equation [52]:

(4) $$S\left(f\right)=A^{2}T_{s}{\left(\frac{\sin^{2}\left(\pi fT_{s}/2\right)}{\pi fT_{s}/2}\right)}^{2},$$

where signal amplitude is noted with *A*, modulation period with *Ts*, and *f* is the frequency. The plotted graph in [52] for Manchester coding shows that a bandwidth of around 20 kHz is needed for a data rate of 10 kbps. This shows that the bandwidths for the band-pass filters in both situations are almost the same. While there are obvious disadvantages related to the BFSK modulation, such as the complexity of the hardware, it is expected to have a better immunity to noise because the central frequency can be chosen in a spectrum region (e.g., at 1 MHz), with a lot less noise from artificial sources.

## 4. Design and Implementation of the Visible Light Communications System with BFSK

**Figure 1:**
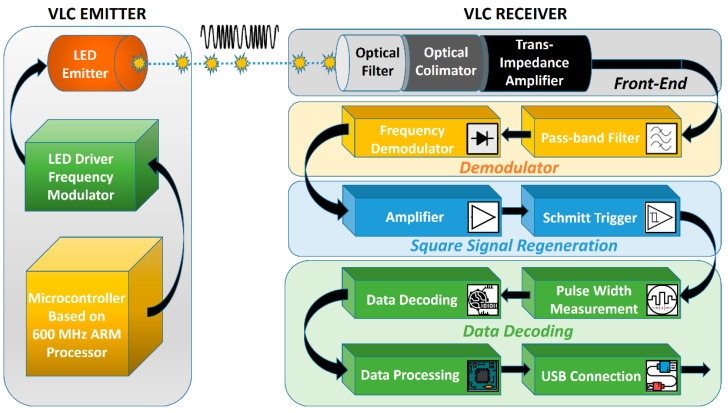
Schematic representation of the VLC BFSK prototype system.

In order to evaluate the opportunity of the BFSK modulation utilization in VLC application and its potential benefits, a new VLC prototype has been designed and implemented. The prototype is formed by two components, the VLC BFSK emitter and the VLC BFSK receiver, as can be seen in Figure 1.

### 4.1. VLC BFSK Emitter

**Figure 2:**
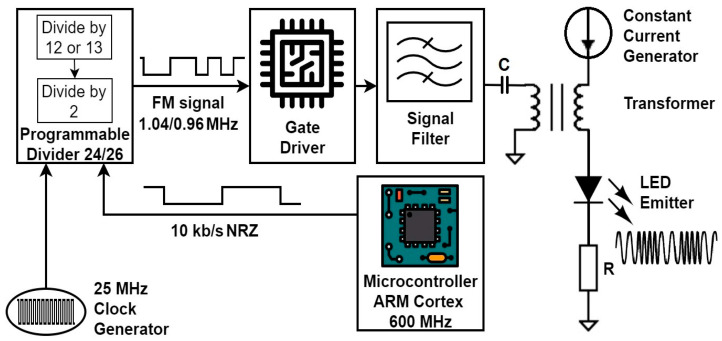
Schematic representation of the VLC BFSK emitter system used in these experiments.

At the core of the VLC transmitter stands a microcontroller board with a 600 MHz ARM Cortex M7 processor, which segments the data and envelopes the data into frames. The chosen code for this setup was the traditional NRZ code, while the data rate was set at 10 kbps. The frame contains a synchronization header and various information such as the data rate and length of the frame. After this step, the raw data are modulated with two frequency signals: the “0”-bit and “1”-bit are, respectively, modulated with 1.04 MHz and 0.96 MHz square signals. These frequencies are generated based on a 25 MHz quartz oscillator (Figure 2), which drives a programmable divider, with division coefficients of 24 or 26, depending on the value of the data bit, resulting in a signal with a duty factor of 50% (Table 1). The final RF driver is based on a TC1411n circuit, which has a switching time of around 25 ns, and the power dissipated in the circuit is small enough to have good efficiency. The TC1411n is a MOSFET driver, having its output in a low-impedance CMOS push–pull configuration.

**Figure 3:**
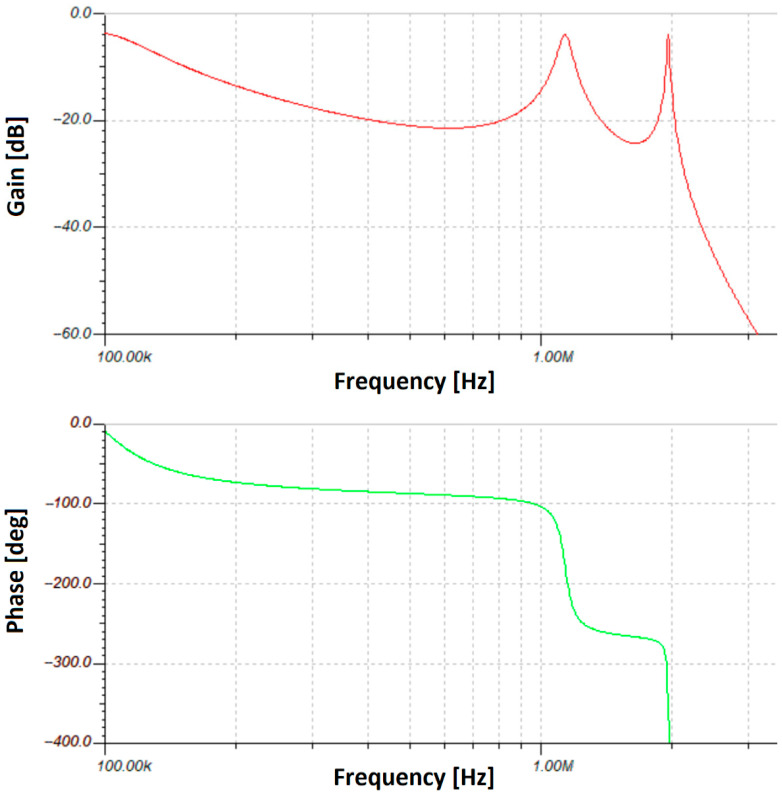
Bode plot for the emitter low-pass filter.

**Figure 4:**
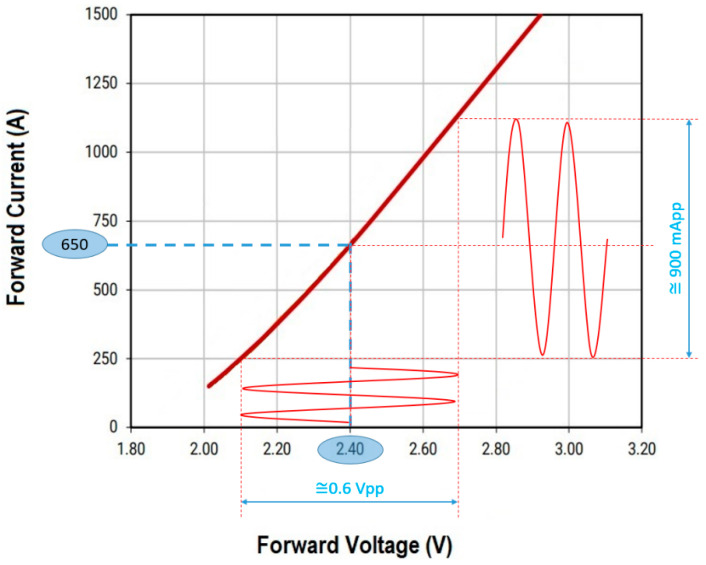
Electrical characteristics curves for XP-E2 LED.

After this stage, a filter is needed to stop the harmonics of the signal so that it becomes sinusoidal. The low-pass filter Bode plot is presented in Figure 3. The LED brightness of the emitter must be modulated around a determined current value. For our experiment, a red LED XP-E2 was used, so an unmodulated current of 650 mA at a voltage of around 2.4 V was chosen based on the datasheet characteristics summarized in Figure 4.

This current is generated by a constant current source made with two transistors. To modulate the brightness of the LED, it is observed from Figure 4 that an RF voltage of about 0.5 V_pp_ sinusoidal signal superimposed over the 2.4 V constant voltage is required. Under these conditions, the filter parameters are determined, as can be seen in Table 1.

**Figure 5:**
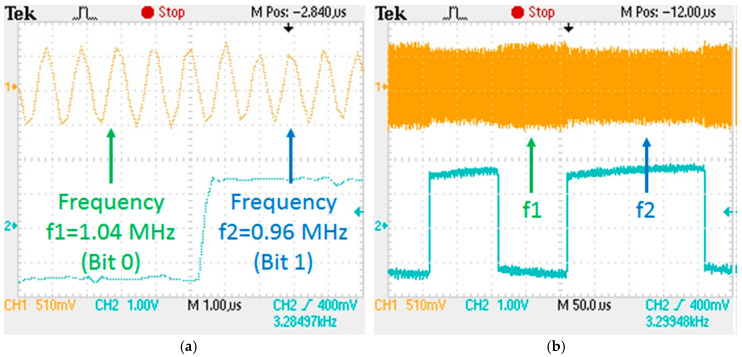
BFSK modulation at the VLC emitter level: (**a**) detailed view; (**b**) general view.

The superimposition of the modulated signal is obtained by adding the constant current generator in series with the output winding of the toroidal transformer. The C capacitor (0.1 μF) blocks the direct current, but offers a low impedance path for the BFSK signal at a frequency of around 1 MHz. The modulated data are converted into light and emitted through the VLC channel. In this configuration, the irradiance of our chosen LED was measured at 1 m distance and it was determined to be around 56 μW/cm^2^. Figure 5 shows an oscilloscope capture, illustrating the data modulation process at the VLC emitter level.

### 4.2. VLC BFSK Receiver

**Figure 6:**
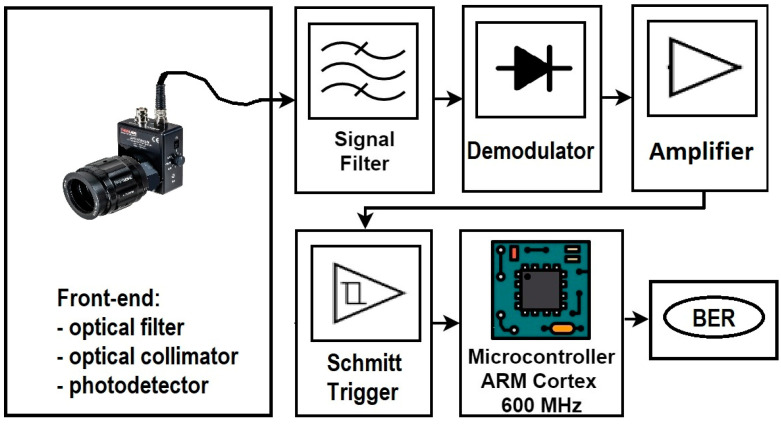
Schematic representation of the VLC BFSK receiver system used in these experiments.

As usual, special attention must be paid to the VLC receiver, as it is the part that has the highest influence on the performance of the entire VLC chain system. The main characteristics are presented in Table 2. In this setup, the VLC receiver has four principal functions: the conversion of light in electrical signals, which is the task assigned to the front-end part, the demodulation, which is carried out by the BFSK demodulator, the signal regeneration, which is realized with the help of a Schmitt trigger module, and the data processing, which is performed by our chosen microcontroller board (Figure 6).

The front-end is fitted with an 80 nm band-pass optical filter centered at 645 nm, which is matched with the wavelength emitted by the XP-E2 red LED. This filter can stop up to 80% of background noise in outdoor settings, improving the SNR. Another component of the front-end is the optical collecting system that limits the receiver’s FoV to ±20°, further improving the SNR, but with a trade-off in mobility. Finally, the most important part of the front-end is the transimpedance amplifier with a PIN photodiode, which offers the necessary voltage levels for the following stages. The PIN photodiode has an 11 MHz bandwidth, and its standard spectral characteristics response is within the 400–1100 nm interval. More exactly, its responsivity is increasing almost linearly with the wavelength increase, from 0.2 A/W at 400 nm, going to 0.5 A/W at 640 nm, and up to a maximum limit of 0.72 A/W achieved at 970 nm, showing a rapid decrease after this point.

The demodulation of the BFSK signal with the frequency of 1.04/0.96 MHz is performed with the help of a non-coherent specialized circuit, KA3361. For our system, the demodulator’s sensitivity was chosen at around 10 mV for a maximum BER value of 10^−6^. In order to increase the immunity to noise, pass-band filters centered at the carrier frequencies are used before the demodulation stage. The resulting signal is regenerated with the help of a Schmitt trigger circuit based on a SN74HCT132 circuit having a slew rate of 8 V/μs, and which will output a digital signal of around 5 V amplitude.

The fourth and final block is a microcontroller board with an ARM Cortex M7 processor at 600 MHz. At this stage, the reconditioned signal is processed in real-time, and the data are extracted asynchronously, with a mechanism of pulse width measurement based on the detection of rising and falling edges. The data are further interpreted with the help of information included in the received header, and BER is calculated by comparing the received bits with the expected sequence programmed in advance.

## 5. Experimental Results

### 5.1. Experimental Procedure and Methods

The main objective of this experiment is to determine if the BFSK modulation and NRZ coding do indeed have an improved immunity to noise compared to the OOK modulation and Manchester coding. In order to determine this, the first step of the experimental procedure is to calibrate our prototype as close as possible to a system with OOK modulation and Manchester coding by conveniently adjusting the gain of their preamplifiers until the irradiance of emitted light has the same value for both modulations. It should be clarified here that the two VLC prototypes, the one based on OOK modulation and the one based on BFSK modulation, are developed based on the same circuits, components, and have very similar software routines. Thus, both solutions use the same platform. On the other hand, some modifications were required in order to enable the adaptation to the BFSK requirements and characteristics. Consequently, different modulation and demodulation circuits are used, as well as different driver circuits at the emitter level, and different band-pass filters at the VLC receiver level, and, of course, different coding and decoding algorithms. Furthermore, despite certain differences, the two prototypes have been calibrated to have rather similar amplification levels and to enable similar communication ranges. In the second step, the aim is to see how both systems behave in response to strong optical noise interference.

#### 5.1.1. The Initial Calibration for the Experiment

In order to have the systems properly calibrated for both OOK and BFSK modulations, two things must be taken into consideration: the irradiance of emitted light must be the same, and the achievable communication distance in laboratory conditions must be the same.

After the necessary adjustments, both VLC systems were calibrated with their respective modulations for the same output irradiance. In order to achieve that, the irradiance was measured through a black tube of 1 m length and 100 mm diameter in order to isolate the ambient noise as much as possible. The output value was set to 56 μW/cm^2^ for both systems, as can also be seen in Table 1.

**Figure 7:**
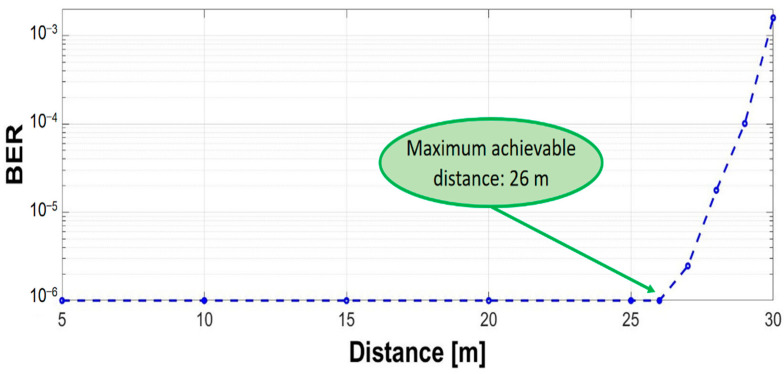
Maximum achievable distance with the VLC BFSK prototype.

Once the irradiance output was established, the next phase of the calibration was to adjust both systems to have the same achievable communication distance with a BER lower than 10^−6^ and a confidence level of 95%. It should be clarified here that although the measurements stopped at the 10^−6^ BER limit, a lower BER would be expected. For this task, the VLC BFSK system was tested in laboratory conditions with natural light. The ambient noise was measured at 10–29 μW/cm^2^ throughout the entire session of measurements and the amplitude of the modulated current was adjusted at 900 mApp, as can be seen in Figure 4. The measurements were made in one meter steps in order to determine the distance at which the BER value exceeded 10^−6^. As is depicted in Figure 7, the maximum achievable distance of communication was determined to be up to 26 m in these predetermined conditions, which was the limit of interest for our comparative system. After that, the gain was adjusted for the VLC system with OOK modulation in order to achieve the same maximum communication under the 10^−6^ BER limit. After this final phase, the calibration of both systems can be considered complete.

#### 5.1.2. The Resilience to Optical Noise Test

In this step of the experiment, the noise resilience of the BFSK VLC prototype has been compared to the one of a VLC system with OOK modulation.

**Figure 8:**
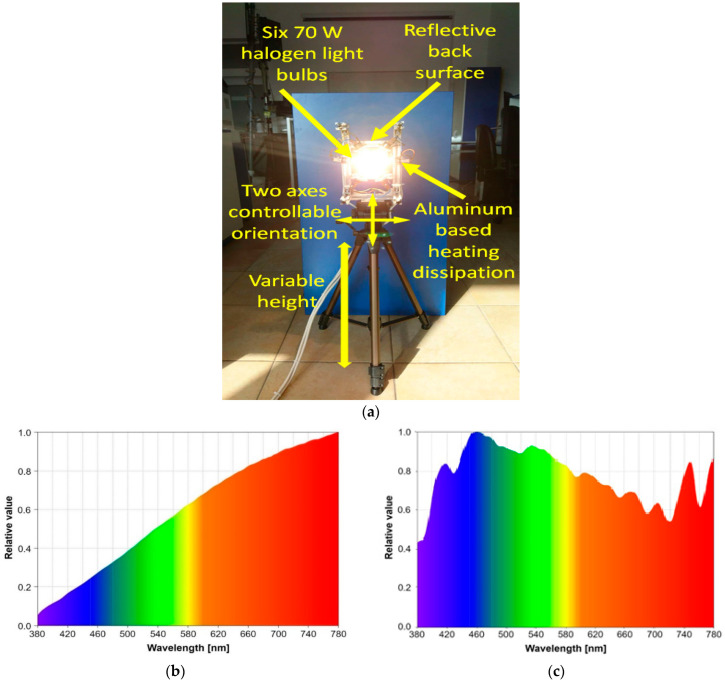
Optical noise source used for these experiments: (**a**) hardware implementation; (**b**) optical noise source spectral analysis; (**c**) example of sunlight spectral analysis within the visible light spectrum.

The parameters used for this scenario are presented in Table 3. Previous experience has shown that such preliminary experiments are less relevant in outdoor conditions where the movement of the sun with respect to the VLC receiver, and the constant modification of the sun irradiance during the day, can lead to misleading results. Furthermore, as shown in [30], the effect of optical noise is strongly influenced not only by its power, but it is also significantly influenced by the type of optical noise (i.e., direct or indirect), and by the incidence angle. Therefore, in order to have higher control, the experiments have been conducted in indoor laboratory conditions. These conditions assumed a minimum amount of indirect sunlight coming through the window and a controlled amount of optical noise. This approach enables us to control not only the intensity of the optical noise but also its location with respect to the VLC receiver photosensitive element (i.e., the light incidence angle with respect to the VLC receiver). Once these aspects were established, the next step was to implement an artificial source of light that could be used in laboratory conditions. For this purpose, six halogen light bulbs having a total power of 420 W were used, being able to generate an optical irradiance of up to ≈28,000 µW/cm^2^ measured in the 400–1050 nm spectral interval, up to 6440 µW/cm^2^ measured in the 400–780 nm spectral interval, and up to 3360 µW/cm^2^ measured in the 600–680 nm spectral interval. For comparison, sunlight in the 400–1050 nm spectral interval can have a maximum irradiance of 65,230 µW/cm^2^, with 47,870 µW/cm^2^ being in the visible light spectrum. Different from the LED white light, which has spectral characteristics that are totally different from the ones of natural light, incandescent light sources emit radiations in all the visible light spectrum, being closer to the sunlight characteristics. Figure 8 illustrates the optical noise source and its spectral characteristics, while also showing a sunlight spectral analysis for the visible light spectral interval.

**Figure 9:**
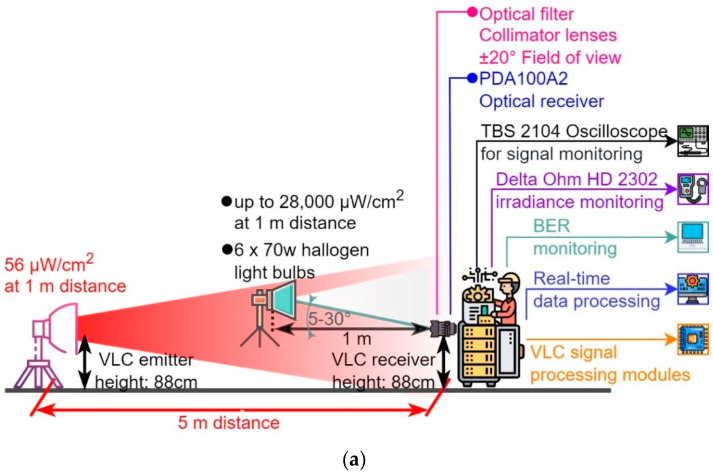

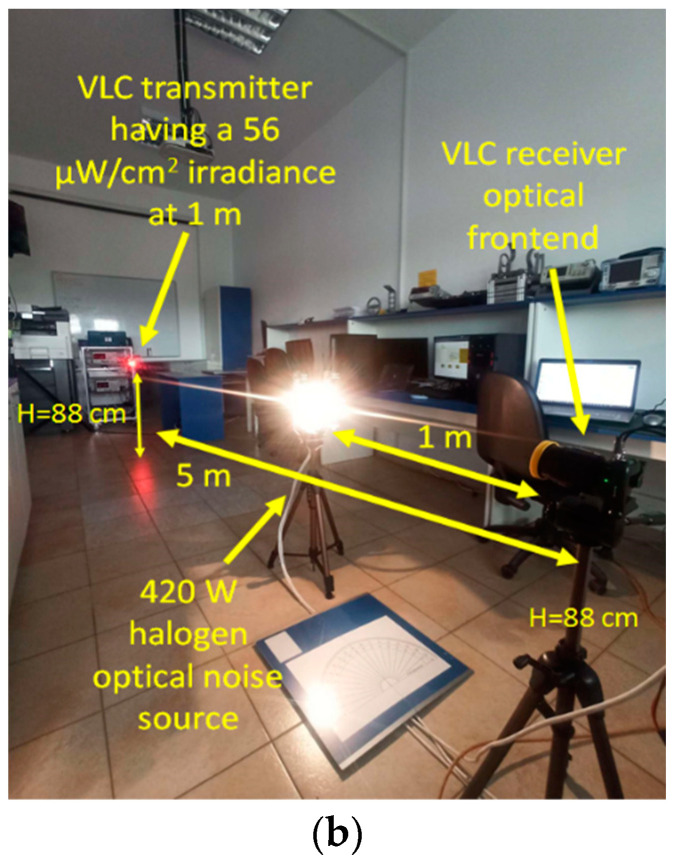
VLC prototype optical noise resilience evaluation: (**a**) envisioned testing setup; (**b**) experimental testing setup.

Next, the emitter and the receiver for each system were placed at a 5 m distance from each other. This distance can represent the range between a high ceiling (i.e., in public places such as museums) and an indoor VLC receiver, but also the distance between vehicles in a platoon. Additionally, as the optical irradiance of the VLC emitter was only 56 µ/cm^2^, whereas the maximum distance determined in Section 5.1.1 was 26 m, it is obvious that if the application imposes it, longer distances are achievable. Nevertheless, as the purpose of this section is to evaluate the noise resilience of the proposed concept, the selection of the emitter–receiver range is less important. Then, in order to have the maximum impact, the optical noise source was placed at 1 m distance from the receiver, whereas the incidence angle with respect to the VLC receiver was around 5° for direct exposure, and around 30° (i.e., outside the FoV) for indirect exposure. Figure 9 shows the envisioned and the experimental testing setup. In these conditions, the maximum BER limit was set at 10^−6^. Once the communication link was established and the BER measurement was started, the source noise power was gradually increased until the maximum BER limit was exceeded.

The resilience to optical noise test took place in two phases: one for the BFSK VLC setup, and one for the VLC with OOK modulation for comparison purposes. The summary of the characteristics of the testing setup used during the experimental evaluation is presented in Table 3, and testing equipment is summarized in Table 4.

### 5.2. Experimental Results in Ambient Optical Noise

This section highlights the experimental results concerning the effect of ambient noise on the VLC BFSK prototype in comparison with OOK modulation. The laboratory testing followed the path described in Section 5.1.

In the first part of the experimental evaluation, the two systems were evaluated in direct optical noise conditions, with the optical noise source being positioned at an angle of 5˚ with respect to the VLC receiver. At this phase of the experiment, the VLC system with OOK modulation and Manchester coding was able to maintain a BER under the maximum imposed limit of 10^−6^ until the optical noise source reached a direct incidence irradiance of ≈2800 µW/cm^2^. After this value, the BER starts to degrade rapidly until the point where the link is lost. In the following part of the experiment, the VLC BFSK prototype was able to maintain its low BER (i.e., under 10^−6^) until the measured optical noise reached ≈3500 µW/cm^2^, which confirmed the hypothesis of a better noise resilience.

**Figure 10:**
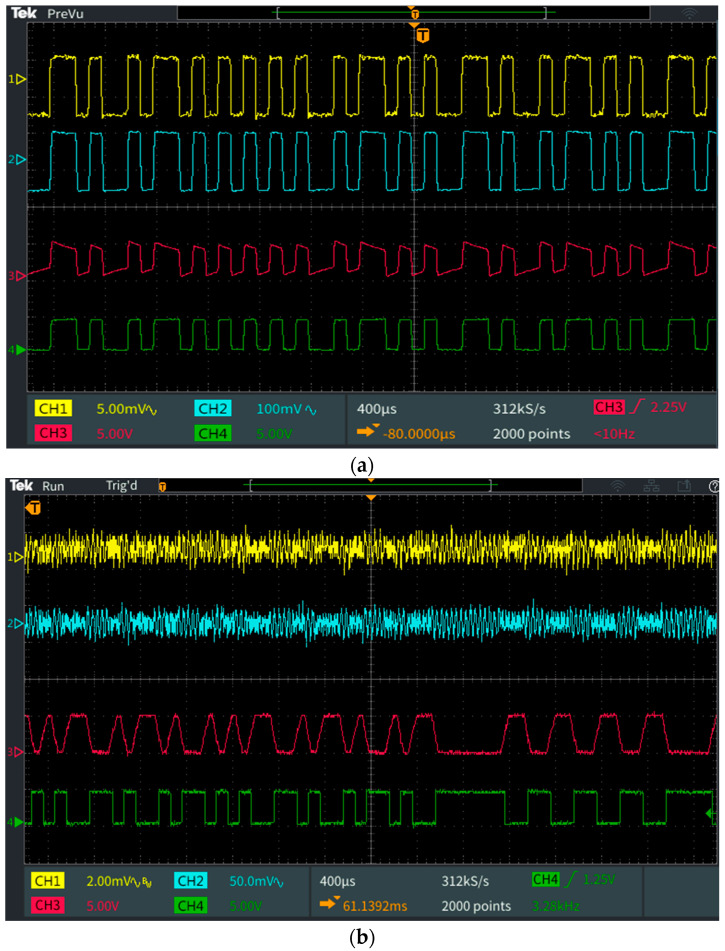
Oscilloscope print-screens showing the signal regeneration process at the level of the different stages of the VLC receiver: Channel 1 (yellow) shows the output of the transimpedance amplifier; Channel 2 (blue) shows amplified Channel 1 signal; Channel 3 (purple) shows the adaptive gain control circuit, and the output of the demodulator, respectively; Channel 4 (green) shows the regenerated signal after Schmitt trigger circuit: (**a**) OOK modulation; (**b**) BFSK modulation.

**Figure 11:**
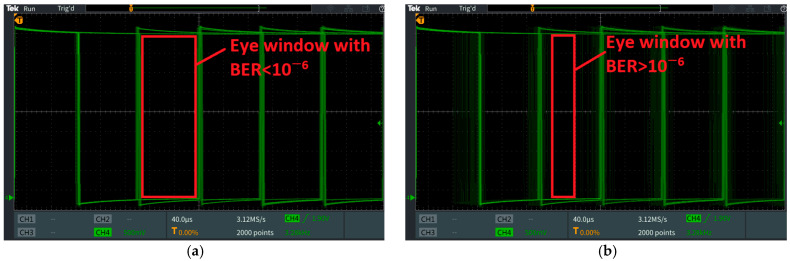

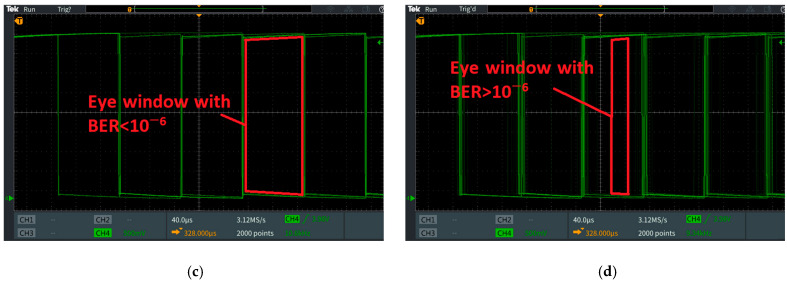
Eye diagrams for OOK and BFSK VLC prototypes evaluation: (**a**) BFSK modulation at ≈3500 µW/cm^2^; (**b**) BFSK modulation at ≈3600 µW/cm^2^; (**c**) OOK modulation at ≈2800 µW/cm^2^; (**d**) OOK modulation at ≈2900 µW/cm^2^.

Figure 10a,b illustrates the signals received by the two VLC prototypes, and their signal reconstruction steps. Figure 10 also points out the difference between the two modulation techniques. Additionally, Figure 11 illustrates the eye diagrams for the two systems, emphasizing the limit where the BER begins to be affected by the optical noise irradiance increase.

Next, in order to measure the resilience to noise in indirect exposure, the VLC system with OOK modulation and Manchester coding was prepared for an incidence angle of 30° from the incandescent source. As soon as the experiment started, it was obvious that the maximum irradiance (i.e., 28,000 µW/cm^2^) of the noise source cannot affect the photodiode in indirect exposure, and therefore it has absolutely no impact over the BER value, which was consistently under the 10^−6^ limit. In order to carry on with the experiment, the decision was made to remove the optical filter in order to expose the receiver to the entire spectrum of the incandescent light, as is shown in Figure 8b. For this, the light spectrum emitted by the noise source was measured with the optical filter fitted on the spectrometer analyzer, and it was compared with the irradiance measured without the optical filter. It was concluded that the optical filter introduces an attenuation with a factor of eight over the entire spectrum. Once this estimation was established, the experiment was started and this time it was possible to determine, for the VLC system with OOK modulation in indirect exposure, that the communication link was able to maintain a BER under 10^−6^ until a noise irradiance of ≈6800 µW/cm^2^, which would be equivalent to an irradiance of approximately ≈54,000 µW/cm^2^ with the optical filter fitted on the receiver. In the second phase of the experiment, the VLC BFSK system was tested in the same conditions, and, this time, the resilience was maintained up to a noise irradiance of ≈8100 µW/cm^2^, which would be equivalent to an irradiance of around ≈65,000 µW/cm^2^ with the optical filter fitted. Table 5 summarizes the results of the entire experimental evaluation.

### 5.3. Debate on the Experimental Results

The experimental results demonstrated that the BFSK modulation has the potential to improve VLC systems’ resilience to optical noise. With respect to the classical OOK modulation, a 20% noise resilience improvement with respect to DC optical noise can be observed. Nevertheless, in addition to the non-modulated interference sources (i.e., the sunlight), modulated optical sources can also affect the performance of VLC systems. As summarized in [9], AC-supplied incandescent light sources generate optical interference having a 100 Hz frequency, with harmonics that can reach up to 2 kHz, whereas fluorescent lamps induce an optical interference of frequencies up to 40 kHz, with low amplitude interference that extends up to 1 MHz. Thus, similar to the approach considered in the IEEE 802.15.7 standard, BFSK use moves the communication to a higher frequency, where less interference can be found, further improving the signal-to-noise ratio.

On the other hand, compared with the classical OOK, the BFSK approach assumes a slightly more complex hardware implementation of VLC emitter and VLC transmitter. Additionally, the BFSK VLC method has a negative impact on the achievable data rate. Thus, as BFSK relies on a sinusoidal optical carrier that must have a significantly higher frequency with respect to baud rate, whereas the LED commutation time is not always as low as would be desirable (i.e., especially in the case of high power LEDs), the use of this method limits the system’s data rate. Moreover, the performance of the system can be also affected by jitter, especially when increasing the data rate. Therefore, although higher data rates are achievable with LEDs that have lower response times, the overall data transfer will always be lower than the one achievable by other modulations such as OFDM or quadrature amplitude modulation (QAM). Even so, although OFDM solutions are more advanced and provide significantly improved data rates, much of the OFDM VLC research is still at a simulation level, with a rather limited number of OFDM VLC prototypes. Consequently, one can admit the superiority of OFDM systems, but one also has to admit that BFSK could be useful in situations where the level of optical noise can rise above certain limits, or in applications in which optical noise resilience precedes data rate.

Table 6 provides a BFSK VLC prototype performance comparison with respect to other VLC hardware systems. As one can see, the proposed concept provides remarkable noise resilience. It should also be clarified that although for simplicity and bandwidth efficiency reasons the NRZ coding has been implemented, it is expected that improved optical noise resilience will be achieved by other coding techniques as well.

## 6. Conclusions and Discussions

In present times, more solutions are necessary for various aspects of modern wireless communications. Beginning with the safety of road traffic and continuing with the special needs for challenged people, the unguided optical communications, especially the VLC systems, could prove to be the solution needed for various challenging scenarios, where RF communications cannot have the expected efficiency. Therefore, this article is focused on the possibility of improving the resilience to noise of VLC systems.

In this context, this article presents a novel idea aimed at adapting the concept of frequency-shift keying modulation to VLC systems. To determine the benefits of the proposed concept and to demonstrate its superiority with respect to the classical OOK modulation, the BFSK VLC system was tested together with an OOK VLC system. Experimental results demonstrated that compared to a classic system based on OOK modulation and Manchester code, a prototype based on BFSK modulation has an enhanced resilience to noise. Thus, the BFSK VLC prototype was capable of sustaining an increase of 25% of irradiance noise in direct exposure, and an almost 20% irradiance increase in indirect optical noise exposure. While this was not the intention of our tests, the important role of an optical filter was nevertheless highlighted throughout the experimental phase under indirect exposure.

Despite the fact that frequency modulation could be a better choice for a VLC system to counteract the artificial noise in a real-life scenario, it should be mentioned that some drawbacks are still present. First, during the calibration session, it was obvious that the current prototype is much more challenging to adjust, which makes it difficult to integrate in more complex systems, such as context-adaptive ones. Another challenge was seen in the test sessions, where the presence of small jitter was seen on the oscilloscope, which indicates that a better design of the system is needed. Although this would not have impacted this experiment, it is possible that there would be some issues at higher data rates. Therefore, in the future, the solutions to these issues should be searched for in order to obtain a truly reliable system, and to validate that this system could indeed be used in real-life scenarios, while studying the influence of the noise in more complex testing scenarios.

## Figures and Tables

**Table 1:** Parameters of the VLC emitter prototype with BFSK modulation.

Parameter	Values
Optical source	XP-E2 (CREE red LED)
Emitted irradiance at 1 m distance	≅56 μW/cm^2^
VLC emitter’s central wavelength	630 nm
Coding technique	NRZ
Data rate	10 kb/s
Encoding hardware	Microcontroller 600 MHz ARM Cortex M7 processor
Oscillator frequency [MHz]	25
Total division factor	24/26
Resulted frequency [MHz]	1.04/0.96
The ratio of the transformer	20:4
Filter inductances [μH]	9
Ferrite rings	T50-1, T50-2
Filter capacitances [pF]	2 × 270, 2 × 2200

**Table 2:** Parameters of the VLC receiver prototype with BFSK modulation.

VLC Receiver Blocks	Parameter	Values/Features
Front-end	Optical filter dominant wavelength	645 ± 40 nm
	VLC optical photodetector	PDA100A2 switchable gain transimpedance amplifier
	Optical collector’s FoV	±20°
BFSK demodulator	Sensitivity	10 mV at a 10^−6^ BER value
Regeneration stage	Square signal regeneration	Schmitt trigger circuit
Data processing	Hardware	Microcontroller board with an ARM Cortex M7 processor at 600 MHz
	Data processing	Rising and falling edge detection and pulse width measurement
	Data decoding	Real-time extraction, with data rate of 10 kb/s
	Monitored parameters	Real-time BER without forward error correcting codes

**Table 3:** Summary of the experimental characteristics.

Parameter	Feature/Values
Testing conditions	Low SNR laboratory conditions
Emitter–Receiver distance	5 m
Natural ambient light	10–29 μW/cm^2^
Modulation technique	Phase 1: BFSK
	Phase 2: OOK
Coding technique	Phase 1: NRZ Phase 2: Manchester
Optical filter	645 ± 40 nm for direct exposure
	None for indirect exposure
Data rate	10 kb/s
Noise source	420 W incandescent light bulbs with a measured irradiance of maximum ≈28,000 µW/cm^2^
Placement of noise source	At 1 m and an incidence angle of 5° for direct exposure
	At 1 m and an incidence angle of 30° for indirect exposure
Measured parameter	Real-time BER determination without the use of FEC protocols, with a 10^−6^ limit

**Table 4:** Testing equipment used during the experiment.

Equipment Type	Model
Irradiance meter	Delta Ohm HD 2302.0 with LP 471 RAD Probe
Optical spectrometer analyzer	Sekonic C-800
Oscilloscope	Tektronix TBS 2104

**Table 5:** Summary of the experimental results.

Parameter	BPSK VLC Prototype	OOK VLC Prototype
Communication range for BER < 10^−6^	26 m	26 m
BER > 10^−6^ limit for direct light exposure at 5 m	≈3500 µW/cm^2^	≈2800 µW/cm^2^
BER > 10^−6^ limit for indirect light exposure at 5 m	≈8100 µW/cm^2^ ≈65,000 µW/cm^2^	≈6800 µW/cm^2^ ≈54,000 µW/cm^2^

**Table 6:** BFSK VLC prototype performance comparison with respect to other VLC prototypes.

Ref.	Modulation/ Coding	Noise Resilience Enhancement Mechanism	BER	Testing conditions	Merits	Disadvantages
[31]	DSSS/SIK	Rather high, provided by DSSS modulation	10^−6^–10^−4^	Bright daytime conditions exposure	High overall performance (range, noise resilience)	The pseudo noise sequence affects data rate
[18,19]	OOK/Manchester	Narrow FOV, optical filters, logarithmic transimpedance circuit	10^−3^–10^−6^	Up to 65,000 µW/cm^2^ sunlight exposure (outside the FoV)	Extended dynamic range and enhanced noise resilience	Amplification is reduced as optical noise increases, limiting the communication range
[21]	OOK/Manchester	Optical lens	3 × 10^−7^	Indoor with artificial light	Standard compliant systems	Insufficient noise resilience
[20]	OOK/Manchester	Optical filtering and SDR processing	3.1 × 10^−7^	12,000 lux daylight exposure	Improved signal processing; 1 Mb/s data rate	
[27]	OFDM	Optical filtering and OFDM	≈10^−3^	50,350 lux exposure	1 Gb/s data rate	Low communication range (14 cm)
This work	BFSK/NRZ	Optical filtering and BFSK	<10^−6^	Up to 65,000 µW/cm^2^ exposure	Higher noise resilience	Low data rate (10 kb/s)

## Data Availability

Not applicable.
